# How Is the Risk of Major Sudden Infectious Epidemic Transmitted? A Grounded Theory Analysis Based on COVID-19 in China

**DOI:** 10.3389/fpubh.2021.795481

**Published:** 2021-11-26

**Authors:** Xin Duan, Zhisheng Zhang, Wei Zhang

**Affiliations:** ^1^School of Management, Anhui University, Hefei, China; ^2^School of Finance and Public Management, Anhui University of Finance and Economics, Bengbu, China; ^3^School of Public Administration, Sichuan University, Chengdu, China

**Keywords:** sudden infectious epidemics, grounded theory, risk transmission mechanism, risk transmission elements, risk transmission path, COVID-19

## Abstract

The outbreak of a sudden infectious epidemic often causes serious casualties and property losses to the whole society. The COVID-19 epidemic that broke out in China at the end of December 2019, spread rapidly, resulting in large groups of confirmed diagnoses, and causing severe damage to China's society. This epidemic even now encompasses the globe. This paper takes the COVID-19 epidemic that has occurred in China as an example, the original data of this paper is derived from 20 Chinese media reports on COVID-19, and the grounded theory is used to analyze the original data to find the risk transmission rules of a sudden infectious epidemic. The results show that in the risk transmission of a sudden infectious epidemic, there are six basic elements: the risk source, the risk early warning, the risk transmission path, the risk transmission victims, the risk transmission inflection point, and the end of risk transmission. After a sudden infectious epidemic breaks out, there are three risk transmission paths, namely, a medical system risk transmission path, a social system risk transmission path, and a psychological risk transmission path, and these three paths present a coupling structure. These findings in this paper suggest that people should strengthen the emergency management of a sudden infectious epidemic by controlling of the risk source, establishing an efficient and scientific risk early warning mechanism and blocking of the risk transmission paths. The results of this study can provide corresponding policy implications for the emergency management of sudden public health events.

## Introduction

In recent years, with the rapid development of the social economy and the unreasonable production and living behaviors of human beings, the relationship between humans and nature has been unbalanced to some extent, and the whole society has faced increasingly more sudden infectious epidemics, such as the SARS epidemic that broke out in 2003 (1), the Ebola epidemic that broke out in West African in 2013 and the COVID-19 epidemic that broke out in December 2019 (2, 3). Due to the sudden occurrence of epidemics, imperfect medical and health emergency management systems and other multiple reasons, once epidemics break out, they often spread rapidly and eventually cause huge losses of life and property to the whole society. The occurrence of COVID-19 has caused a huge impact on the global economy, and the global economy is facing tremendous pressure of recession, for example: the global flow of people and logistics caused by the epidemic has been forcibly cut off, people cannot flow freely, the production and operation activities of enterprises have stagnated, and business confidence has fallen. In addition, the COVID-19 pandemic has infected thousands of people. The outbreak of COVID-19 has resulted in more than 80 thousand confirmed cases in China and a death toll exceeding 4,500 (4). In the face of the COVID-19 pandemic that is spreading around the world, how to mitigate the negative impact of COVID-19 has become a topic of widespread concern. Why does the outbreak of sudden infectious epidemics often cause huge social losses to human society? The key to answering this question is to clarify the risk transmission mechanism of sudden infectious epidemics. There have not yet been any scientific answers to the question in academic circles. Clarifying the risk transmission mechanism of sudden infectious epidemics can help us understand the risk transmission rules of sudden infectious epidemics and can then provide policy implications for the efficient crisis management of sudden infectious epidemics. Therefore, this paper uses Chinese media reports on COVID-19 as data, and uses grounded theory to analyze these data to find the risk transmission mechanism of sudden infectious epidemics, which mainly includes the risk transmission elements and risk transmission process.

The content of this paper is as follows. The first part of this paper introduces the research background and purpose. The second part of the paper reviews the relevant researches about risk transmission, puts forward the definition of risk transmission and analyzes the status of the academic community's research on the outbreak of sudden infectious epidemics. Then, the third part of the paper introduces the research methods, and data sources, as well as the epidemic case. Next, the fourth part of the paper presents the research process, namely, the process for coding the data through the use of grounded theory, including open coding, axial coding, selective coding and saturation tests. In the fifth part, this paper comprehensively explains the risk transmission mechanism model of sudden infectious epidemic. Last, the paper summarizes the main findings of the research, then, policy suggestions, research limitations and prospects are provided.

## Literature Review

### The Definition of Risk Transmission

An extensive reading of related literature reveals that the concept of risk transmission has been widely used, especially in the medical health field. For instance, Hayama et al. (5) used foot-and-mouth disease (FMD) transmission model to evaluated the transmission risk of foot-and-mouth disease (FMD) in Japan. Cottrell et al. (6) found avoidance of breastfeeding does not seem to be indicated for reducing transmission risk of hepatitis C virus. In addition, Zeng et al. (7) analyzed the conduction channels and intensity of health risks caused by haze. In these studies, risk is considered a harm, or a negative effect, based on the above research on risk transmission and combined with the huge loss to society caused by COVID-19, the paper assumes that the essence of risk transmission is a diffusion process with negative influences and harms. In this paper, the harm of sudden infectious diseases is not only limited to the diagnosis of patients, but also involves people's psychological panic and other aspects. It is not clear how the risk of sudden infectious disease transmits, so the purpose of this study is to reveal the risk transmission rules of sudden infectious diseases. In other words, this study takes the COVID-19 outbreak in China as an example to analyze the process of the risk transmission of sudden infectious diseases. Only by understanding the risk transmission process of sudden infectious diseases can we better deal with it.

### Relevant Research on Sudden Infectious Epidemics

A survey of the literature shows that the current research studies on public health epidemic control are numerous and that these studies have been mainly carried out from the following two aspects. On the one hand, these studies have been conducted from the perspective of the epidemic itself, its genetic characteristics (8), epidemic characteristics (9), and etiological diagnosis (10). Moreover, the corresponding diagnostic drugs and treatment methods of the epidemic virus have been analyzed (11, 12). For example, Gire et al. (13) used genome monitoring to clarify the virus origin and transmission process of Ebola and found no evidence of other zoonoses. In general, the main purpose of these studies was to deepen the understanding of the epidemic virus and epidemic spread and to identify corresponding diagnosis and treatment methods and diagnostic drugs in order to fundamentally curb sudden infectious epidemics. On the other hand, from the perspective of public health, studies have analyzed the decision support systems (14), economic loss (15), information network systems (16), crisis management, and other related aspects (17). For example, taking SARS as an example, Quah and Hin-Peng (18) conducted a telephone interview with adults in Singapore and found that gender, age, and attitude, affect the preventive measures taken by the public. In addition, Sands et al. (19) pointed out that, due to insufficient investment in the preparation and response to sudden infectious epidemic crisis, people seldom consider the risk of potential epidemics in macroeconomic prediction. The authors proposed a method to assess the economic vulnerability of different countries and world regions suffering from the spread of sudden infectious epidemics. In general, the research in this area has focused on the emergency management and risk assessment after the outbreak, the improvement of the emergency management ability of the whole society to respond to the outbreak and the reduction of the damage caused by the spread of the epidemic to human society.

These research results are helpful for improving people's understanding of the outbreak of sudden infectious epidemics and for improving the emergency management of sudden infectious epidemics. However, the sudden outbreak of an epidemic from outbreak to control is a process of the continuous evolution of the epidemic, the continuous spread of risks and the people's constant adjustment of response strategies. The people's response strategies often undergo a transition from passive response at the beginning of the outbreak to active and active control. Therefore, it is very important to analyze the mechanism of risk transmission. A sorting out of the literature, revealed that the current research on the risk transmission of sudden infectious epidemics is still in a relatively nascent stage. Consequently, from the perspective of risk transmission, to promote the theoretical research on sudden infectious epidemics, this paper uses grounded theory to study the risk transmission mechanism of the sudden infectious epidemic by drawing evidence from the COVID-19 epidemic in China.

## Research Method and Case Introduction

### Research Method and Data Sources

As its research method, this study adopts the grounded theory proposed by Barney Glaser and Anselm Strauss in 1960s (20). Grounded theory supports exploratory research, emphasizes the natural emergence of research problems and theories, and build theories through standardized and rigorous research procedures (20). As a method of mining theory, the basic steps of grounded theory are as follows. First, raw materials are collected. Second, using the research questions, the concept, and category are summed up from the original materials through a coding analysis. Third, the main category is determined according to the relationship among the subcategories, and the influencing factor model is constructed to explain the connection subcategories. Fourth, the related theory is summarized, and the reserved data is used to test the theoretical saturation. Finally, a summarization and conclusions are made (21). Note that the most important step in the application of grounded theory is the step-by-step data coding, including open coding, spindle coding, and selective coding (22). This method has strong applicability for the study of the risk transmission mechanism of current sudden infectious epidemics. The study's data sources are all mainstream media and famous news websites in China. From February to April 2020, Chinese media reported a large number of news about COVID-19. Journalists report on the epidemic comes from interviews with officials, doctors, community workers and experts, as well as their observations, so these news reports actually provide a wealth of data for us to analyze the risk transmission of COVID-19. Specifically, the sources include *Sourthernweekly, Sanlian Lifeweek, China News, Xinhuanet, Caixin.com*, www.thepaper.cn, and www.guancha.cn. The criteria and basis for selected news reports were that the three authors read the news reports separately, and then only selected reports about the COVID-19 itself. A total of 20 news reports about COVID-19 were extracted from the above sources to conduct the research (see Appendix).

### The Introduction of the Case

**Figure 1:**
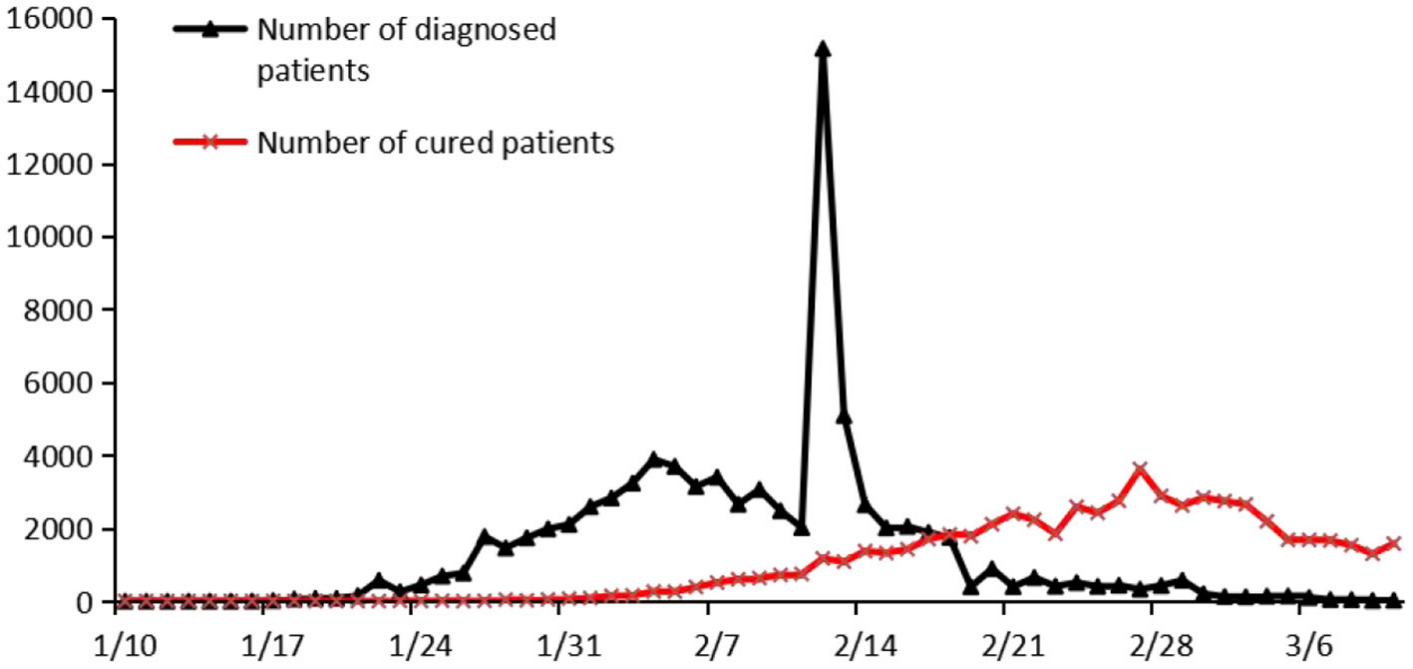
Daily number of diagnosed patients and people cured in China.

Since the end of December 2019, a surveillance of influenza and related diseases had been continuously carried out in Wuhan, Hubei Province. A number of cases of viral pneumonia were found, all of which were diagnosed as viral pneumonia or pulmonary infection. Most of the infected patients in the early stage of the outbreak had contact experience with the seafood market in South China. However, due to the medical staff's limited knowledge of the COVID-19 virus, the conclusion that the epidemic could be controlled and would not infect other people was reached at an early stage. However, with the rapid spread of the epidemic, the number of people infected increased dramatically. On the evening of January 20, 2020, Nanshan Zhong, who was guiding the work of epidemic prevention and controlling, confirmed the human transmission of the disease. Since then, Wuhan has introduced the “closure” measures. On January 27, 2020, Premier Keqiang Li of the State Council came to Wuhan specifically to investigate and guide the epidemic prevention and control work and to visit patients and the medical staff. The epidemic of COVID-19 was declared a public health emergency of international concern (PHEIC) on the night of January 30, 2020 by the World Health Organization (WHO). The number of COVID-19-diagnosed patients had reached 80,859 cases in China as of March 7, 2020, and 50 thousand people had been cured (see Figure 1). The COVID-19 has caused a total of 5,696 deaths in China, and has had severe negative impact on China's economy. In the first quarter of 2020, China's gross domestic product (GDP) grew by −6.8%, while Hubei province's gross domestic product (GDP) grew by −36.75%. In addition, there have been numerous factories shutdowns, traffic suspensions, and schools opening delays. The Chinese government has urged people to reduce travel and congregate to deal with the outbreak. In response to the outbreak in Hubei, the Chinese government has mobilized a large number of medical personnel from other provinces and cities to support Hubei. In addition, many scientists are actively developed vaccines to deal with the epidemic. After the vaccines are developed, the Chinese government encourages people to get vaccinated, and all vaccinations in China are free. As of 30 October 2021, the total number of novel coronavirus vaccine doses in mainland China, Hong Kong and Macao, had reached 268.362 million.

## Research Process

### Open Coding

Initial coding is also called open coding. Its main process involves breaking up the original data and then condensing the relevant data through continuous comparisons and classification; finally, the data are conceptualized (23). The data for this study are from 20 reports on COVID-19 from mainstream media and news websites in China (see Appendix). Following the data analyze process of initial coding, this study first marked all sentences of 20 reports and then deleted sentences with similar, same and unclear meanings. Finally, the remaining sentences after deletion are classified into 11 categories (see Table 1). Table 1 shows the original concepts and categories resulting from open coding.

**Table 1:** Examples of open coding analysis.

**NO**.	**Categories**	**Original statements**
1	Source of virus	A1. Wuhan Municipal Health Commission unexplained pneumonia emergency notice started.
		A2. The Shi Zhengli team of the Wuhan Institute of Virology, Chinese Academy of Sciences, also announced on this day that the source of the COVID-19 is likely to be a bat.
		A3. It might be an artificial virus leaked from the laboratory.
2	Early warning of epidemics	B1. On December 31, 2019, the relevant departments established a notification system for the progress of the epidemic, but in the contents of the notifications, there has been no “14 medical staff infected” information, and notifications have always stressed that the epidemic can be prevented and controlled.
		B2. Experts believe that COVID-19 infection risk may continue to increase but that the epidemic can still be prevented and controlled.
3	Casualties of medical staff	C1. Contracting COVID-19 pneumonia, Liu Zhiming, President of Wuchang hospital, died in Tongji Hospital.
		C2. On February 13, the National Health Commission reported 1716 confirmed cases and 6 medical staff deaths. Four days after this figure was released, the number of medical staff killed working in the front lines of the anti-epidemic was updated to 8.
4	Shortage of medical materials	D1. From New Year's Eve to the first day of the Lunar New Year, many hospitals in Wuhan are in urgent need of materials consisting mainly of protective clothing, masks, goggles, etc.
		D2. They went to the dental room of the clinic, refitted the mask and put it on their forehead. The protective clothing is in short supply. Everyone was only given one piece of protective clothing and has been wearing it for several days.
		D3. My father received the admission form in the hospital, but he cannot get in due to the short supply of beds in the hospital. In the hallway of the hospital, people are positioned close to each other.
5	Human infection	E1. COVID-19 pneumonia was confirmed in Suizhou, with 641 cases as of February 3rd; the 641, number represents an increase of 183 cases, an increase of nearly 40% compared with that of the previous.
		E2. In terms of the diagnosis rate, the ranking of cities has changed: Wuhan is first, Ezhou is second, and Suizhou is third. Of every 10,000 people in Wuhan, ~589 cases were diagnosed; of every 10,000 people in Ezhou, ~308 cases were diagnosed; of every 10,000 people in Suizhou, ~2.9 cases were diagnosed.
		E3. 00:00–24:00, April 7, 2020. In Heilongjiang Province, 1 case of an overseas cured discharge, 2 cases of imported severe cases, and 25 cases of imported confirmed cases were added.
6	Psycholo	F1. The COVID-19 pneumonia epidemic has caused some tension in the Spring Festival.
	gical nervous and panic	F2. A large number of medical workers wearing protective clothing and doused with sprayers were sterilized in the market. It is similar to the situation that occurred when SARS occurred in 2003, and aroused panic.
		F3. Some patients with emotional instability have developed fear, anger, and even despair after diagnosis and have torn the doctor's protective clothing.
7	Psychological exclusion	G1. Phrases referring to many people from Wuhan have changed from “tourists to unwelcome Wuhan people.”
		G2. Some places have labeled and demonized Wuhan people, and even some people returning from Wuhan cannot avoid this situation.
		G3. Hubei people are our compatriots, not monsters.
8	Social production affected	H1. Due to the COVID-19 pneumonia situation, we should implement some tax and fee reduction policies for tourism, catering, hotels, civil aviation, transportation, and life service enterprises, which are more affected by the new crown-like pneumonia epidemic situation.
		H2. Due to the employees' extended working hours because of the epidemic, the company needs to deal with them regarding paid leave and other issues. Therefore, the enterprise bears the corresponding costs. For these costs, the government can moderately allow the enterprise to fully account for them as expenses before income tax.
9	Social life affected	I1. A decision was made to cancel marriage registration at 17 city and district levels; workers were originally scheduled to work overtime on February 2 (Sunday) to handle the marriage registration for new couples.
		I2. The designated hospitals were overcrowded, and some outpatient clinics and wards of general hospitals were closed to prevent the spread of the epidemic. How can some special patients, such as pregnant women, with infection or suspected infection obtain timely diagnosis and treatment?
		I3. February 17 was supposed to be the opening day of primary and secondary schools in many parts of the country. The noisy playground was empty.
10	Inflection point of epidemics	J1. Even the most optimistic experts dare to say that the expected turning point of the epidemic is expected on February 8.
		J2. American scholars predict that the turning point of pneumonia in Wuhan may be delayed to the end of February.
		J3. According to the latest data, on February 17, 79 newly confirmed cases were found in areas other than Hubei, which has seen a two-week decline.
11	The epidemic disappeared	K1. At 0–24:00 on March 11, 2020, Anhui Province reported no new confirmed cases and no new suspected cases.
		K2. As of March 26, 2020, no new confirmed cases have been found in Hunan Province for 25 consecutive days.
		K3. No COVID-19 pneumonia was confirmed in any cases from 00:00 to 24:00 on March 27, 2020, and no imported cases were found.

### Axial Coding

Axial coding is a process comprising the following activities: further refining, adjusting and classifying the various categories obtained from open coding; combining parts with similar or similar meanings; and clarifying and combining the internal relations subcategories (24). As sudden infectious outbreaks tend to undergo a process of occurrence, spread, and extinction, based on this consideration, the 11 categories in Table 1 were refined and classified to obtain six main categories (see Table 2).

**Table 2:** Axial coding.

**Main category**	**Subcategory**	**Connotation of categories**
Risk sources	Source of virus	According to its source, a virus can be divided into a natural source or a human-made source. A virus from a natural source refers to a virus existing in nature but that can spread due to uncontrollable elements, such as human production activities or diet activities. A human virus refers to viruses created in the laboratory and leaked out due to human factors.
Risk early warning	Early warning of an epidemic	This category refers to the epidemic's scientific early warning transmitted through a risk early warning system and that at the beginning of a sudden epidemic's outbreak, is initiated in order to prevent the spread of the epidemic.
Risk transmission path	Shortage of medical materials Medical staff casualties Social production activities affected Social life affected Social psychology affected	The risk transmission path of an infectious epidemic refers to the continuous spread of the risk caused by the epidemic along the risk transmission path; its harm can continuously cause people to lose their lives and property through the risk transmission path.
Risk transmission victims	Casualties Social production activities affected Social life affected Social psychology affected	The scope of the victims of the epidemic is very wide, and includes doctors, nurses, patients and many other social groups. To be more specific, the epidemic caused work stoppages at corporations, delays in school openings, etc. For example, the COVID-19 epidemic has caused many enterprises to stop production and students to postpone attending school. In a way, they are all victims of the epidemic's risk transmission.
Inflection point of risk transmission	Inflection point of the epidemic	The inflection point of an epidemic's risk transmission refers to the time point when along with the comprehensive promotion of epidemic emergency management measures, the total number of confirmed cases gradually decreases and the number of new patients starts to decline.
End of risk transmission	Epidemic disappeared	The end of risk transmission epidemic risk means that the epidemic situation is under full control, is no longer spreading, and human society can fully resume production and lifestyle activities.

### Selective Coding

**Figure 2:**
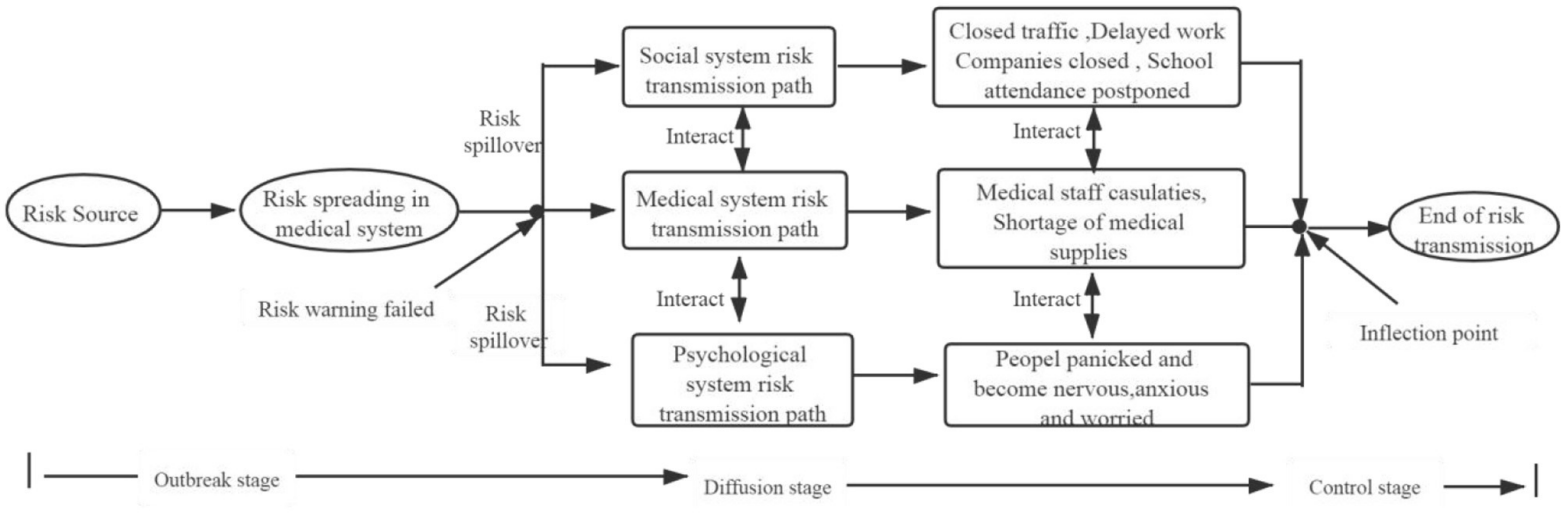
Risk transmission mechanism model of sudden infectious epidemics.

Selective coding comprises the following activities: digging out the core category; then analyzing the relationship between the core category and other main categories; describing the relationship between the core category and the main category in the form of a “storyline”; and, finally, developing the corresponding theoretical results (25). The typical relationship structure of the six main categories formed by spindle coding has been developed (see Table 2). Through analyze and research, the “risk transmission mechanism of sudden infectious epidemics” was determined to be the core category. Due to the sudden outbreak of infectious disease generally have to go through the process of occurrence, spread and extinction. Based on the perspective of the occurrence, spread and extinction of sudden infectious diseases, this study combined the relationship between the six main categories and core categories obtained by the principal axis coding, a risk transmission mechanism model for sudden infectious epidemics was developed (see Figure 2). In other words, a theoretical framework for analyzing the risk transmission rules of sudden infectious epidemics is presented.

### The Test of Theoretical Saturation

Theoretical saturation refers to the moment when additional data cannot be obtained to enable the analyst to further develop the characteristics of a certain category (26). Using other news reports arranged in this article as the original text, and labeling, coding, and conceptualizing them, no obvious novel initial concepts, categories, and relationships were found. This indicates that the “risk transmission mechanism of sudden infectious epidemics” model has an appropriate theoretical saturation (see Figure 2).

## Results

Why do sudden infectious epidemics often cause great damage to human society? An important reason lies in the lack of scientific understanding of the law of risk transmission. This paper draws evidence from the reports of COVID-19 in Chinese media, uses grounded theory to clarify the risk transmission mechanism of sudden infectious epidemics (see Figure 2) and to provides a framework for understanding the risk transmission rules of a sudden infectious epidemic. Then, this article explains the risk transmission mechanism model of sudden infectious epidemics from two analytical aspects: the risk transmission elements and the risk transmission path.

### Risk Transmission Elements of Sudden Infectious Epidemics

The risk transmission of sudden infectious epidemics refers to the process in which the virus spreads continuously with the flow of people and causes serious human society damage due to the failure of epidemic early warning and the failure of timely emergency management. The main manifestations of these damages are casualties, the shutdown of related industries, social panic, and even turmoil. With the continuous spread of sudden infectious epidemics and the emergency management of society, the risk of sudden infectious epidemics presents the dynamic characteristics of continuous evolution, and the epidemic virus also have the possibility of variation in its spread. Table 2 shows that in the whole process of risk transmission of COVID-19, there are six elements, namely, risk sources, risk early warning, risk transmission paths, victims of risk transmission, the risk transmission inflection point, and the end of risk transmission. These six elements will be analyzed in detail as follows.

#### Risk Sources

The virus is the risk source of the outbreak. According to its causes, an epidemic virus can be divided into two different types: a natural risk source or a human risk source virus (27). However, the causes of the COVID-19 epidemic in China are unknown; only the genome of the virus has been sequenced. These results show that COVID-19 and SARS-CoV have a homology of 75–80%, and the genetic relationship of COVID-19 to a bat virus is greater (28). With the rapid development of human society, the relationship between human and nature is out of balance. People need to pay attention to the outbreak of viruses in nature caused by human production and other activities. In addition, climate change has been found can lead to the introduction of some sudden infectious epidemics into previously unaffected geographical areas (29). It is thus very important to strengthen the response to global climate change. At the same time, it is important to strengthen the management of medical biological laboratories to prevent experimental virus leakage.

#### Risk Early Warning

At present, in order to reduce the damage caused by sudden infectious epidemics to human society, people have focused on building various early warning systems and models (30, 31). For instance, Lowe et al. (32) predicted the risk of the mosquito-borne disease dengue fever during the period of the 2014 World Cup in Brazil. The probability prediction of dengue fever risk in 553 Brazilian microregions, comprising the 12 cities identified by the risk level warnings and in which athletes would be competing, was used to solve the possibility of dengue fever prevalence during the championship.^.^ Connor and Mantilla believed that interannual climate change was an important determinant of epidemics in parts of Africa and that climate has promoted both the transmission of mosquito vectors and the incidence of parasites. Therefore, they discussed the use of Europe's leading global ocean-atmosphere coupled climate model that would be dynamically based on seasons (33). In this COVID-19 epidemic in China, the lack of comprehensive knowledge of the epidemic virus by medical experts led to the conclusion that in the early stage of the outbreak, it was preventable and controllable. In addition, at that time, no human-to-human transmission and no medical staff infection had been identified. The failure of early warning of the risk of the epidemic has caused further epidemic transmission.

#### Risk Transmission Path

The risk from sudden infectious epidemics not only comprises the fact that the epidemic can cause casualties in diagnosed patients but also the fact that it can cause damage to the mental health of social staff and the economic life of society (34–36). The risk caused by the outbreak was initially concentrated on the diagnosed patients and medical staff. However, due to the failure of the early warning of the outbreak, the risk of the outbreak continued to spread along the risk transmission path, and the harm caused by the epidemic also continued to expand. Therefore, this paper finds that the epidemic risk transmission path refers to the process of damage caused by the spread of the epidemic to human society. Blocking the spread of the epidemic is an important way to block the risk transmission and reduce the casualties and property losses (37). The initial outbreak of COVID-19 occurred in Wuhan, Hubei Province, and the traffic accessibility of the nine provinces of Wuhan City and the large-scale movement of people during the Spring Festival period led to the rapid spread of the COVID-19 epidemic. Therefore, in order to curb the spread of the epidemic and reduce the damages, on the morning of January 23, 2020, Wuhan adopted a “closed city” measure that prohibited people from entering and exiting and advocated that the citizens maintain social distance and reduce staff gathering activities.

#### Victims of Risk Transmission

The social impact caused by a sudden infectious epidemic can not only be manifested in casualties but can also be reflected in its effect on people's mental health and other industries in society (38–41). For example, Kim et al. (42) analyzed the impact of SARS on the Korean hotel industry. Their study found that compared with the same period in the previous year, the hotel occupancy rate in South Korea decreased by nearly 14% from February 2003 to July 2003. The impact of SARS on the Korean hotel industry was actually more harmful than the 9/11 terrorist incident (42). Research by Novelli et al. (43) asserted that health-related crises in developing countries might affect the tourists' perception of risk, and could therefore result in a sudden drop in tourism demand and could have a major impact on the socioeconomic status of tourism-dependent countries (43). The outbreak of the COVID-19 epidemic in China has caused many people to become infected, many companies to cease operations, and schools to delay their opening. To a certain extent, they are all the victims of this outbreak. The social damage caused by the COVID-19 epidemic is affected by factors such as the nature of the virus, the speed of the infectious epidemic and the emergency management of it in the entire society. In addition, in this epidemic, this paper found that during the transmission of COVID-19, the patients infected with the COVID-19 were both the victims of the epidemic risk transmission and the risk source of the new epidemic transmission. Therefore, in the emergency management of the COVID-19 epidemic, Wuhan rushed to build the “Vulcan Mountain” and “Raytheon Mountain” hospitals and established a large number of square cabin hospitals to isolate a large number of infected and suspected infected patients.

#### The Risk Transmission Inflection Point

A sudden infectious epidemic will undergo an epidemic process (44). At present, many scholars have launched prediction studies on the inflection point of the transmission of COVID-19 in China. If there is an incremental decrease in 2 consecutive days, this indicates that a new trend has occurred, according to this basic principle, Gu et al. (45) suggest that the inflection point of the COVID-19 epidemic may have passed. According to the daily data of newly diagnosed cases, the inflection point of China's COVID-19 epidemic was inferred to have occurred on February 9, 2020 (45). Peng et al. (46) estimated that by mid-March, the work of epidemic prevention will have made much progress in the majority of China's regions, even including Hubei Province, the most serious regions infected by 2019-COVID epidemics. They expected that the epidemic's inflection point in Wuhan would occur in early April (46). In this paper, the inflection point of epidemic risk transmission is considered to refer to the time point at which along with the comprehensive advancement of epidemic emergency management measures the number of new patients is gradually reduced and the total number of cumulative diagnoses starts to decline. In this COVID-19 epidemic, to realize the early arrival of the inflection transmission point of epidemic risk, it is necessary for the Chinese government, enterprises, and the public to coordinate their activities and to take comprehensive measures to jointly promote the emergency management of the epidemic in order to block the epidemic's infection path and reduce the number of newly diagnosed patients. In dealing with the COVID-19 epidemic, the Chinese government has taken strong emergency measures. The National Health Commission stated on March 13, 2020 that the epidemic peak had passed. After the risk transmission inflection point, the Chinese government has taken many measures, such as encouraging people to wear masks and go in and out of public places to take their temperature, to prevent the number of people infected by the epidemic from rebounding. Finally, to end the risk, researchers in various countries are working on vaccines to end the risk of COVID-19.

#### End of Risk Transmission

The disappearance of the transmission of epidemic risk means that the epidemic is under comprehensive control, will not continue to spread and human society can fully resume production and lifestyle activities (47, 48). According to the latest COVID-19 epidemic report, the epidemic was predicted to be basically controlled by the end of April 2020, and by that time, people would not have to wear masks in daily life, and social life would be fully restored. The COVID-19 epidemic is spreading abroad. Therefore, preventing the import and transmission of confirmed cases is the focus of current epidemic prevention and control in China. In the outbreak of SARS in China in 2003, the SARS epidemic did not gradually disappear until the middle of 2003, a period that could therefore be regarded, as the one denoting the end of risk transmission.

### Risk Transmission Path of Sudden Infectious Epidemics

Based on the above analysis of risk transmission elements of sudden infectious epidemics, the following will focus on the transmission path of epidemic risks. The risk of a sudden infectious epidemic is the fact that it cannot only cause casualties in the diagnosed patients but can also damage the mental health and the economic life of society (49–51). Therefore, it is important to analyze the risk transmission path of sudden infectious epidemics. Based on the main category and sub-categories formed by the main axis coding in Table 2 and the study of the hazards of sudden infectious epidemic by scholars (52–54), the risk transmission path of sudden infectious epidemic can be divided into medical system risks transmission path, social system risk transmission path and psychological system risk transmission path (see Figure 2).

**Figure 3:**
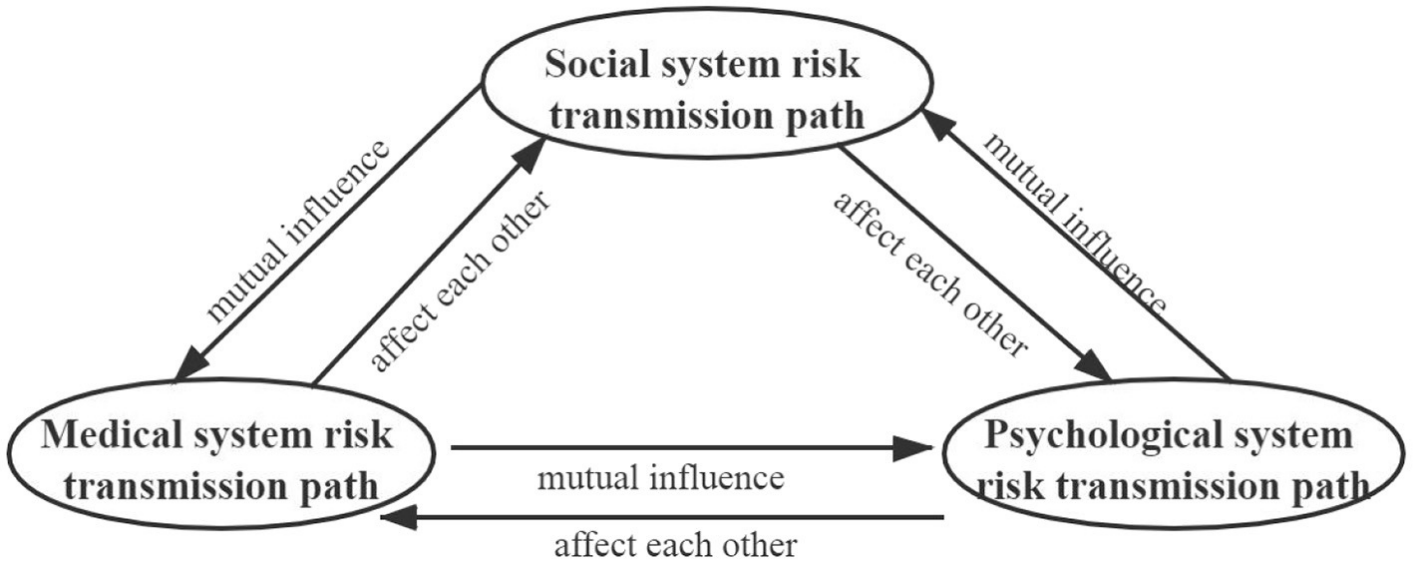
Coupling structure of the epidemic risk transmission path.

In China's outbreak of COVID-19, this paper found that the risk of the epidemic was initially transmitted within the medical system and infected the health care workers. However, because of the failure of risk early warning system and the lack of awareness of the epidemic, the epidemic spread quickly to the whole society. Specifically, this has led to a gradual increase in the number of confirmed patients, the development of psychological problems in people, and the cessation of production by enterprises. Ultimately, the medical system risk transmission path, the social system risk transmission path, and the psychological system transmission path were formed (see Figure 2). The above mentioned three paths of epidemic risk transmission show a coupling structure with interactions and mutual influence (see Figure 3).

#### The Risk Transmission Path of the Medical System

The medical system is a pioneer in blocking the spread of epidemic risk and bears the responsibility of providing scientific early warning and treatment of the epidemic. The failure of the emergency management of the medical system directly lead to the risk's further spread, which further aggravate the damage caused by the epidemic, to people's lives, health and property. The risk transmission path of the epidemic medical system refers to the sudden infectious epidemic's continuous spread in the medical system, in which the spread is due to multiple reasons, such as an insufficient understanding of sudden infectious epidemics, insufficient medical staff and medical supplies, and new infections of diagnosed patients and medical staff. In the early stage of the development of the COVID-19 epidemic, the health and anti-epidemic system made a mistake in its judgment: it was concluded that the epidemic was preventable and controllable and that it would not be transmitted from person to person. However, the spread directly caused the infection of medical staff. As of February 18, there were at least 9 medical staff members who died of confirmed infections. Due to the limited knowledge of the COVID-19 epidemic and the lack of emergency protection, the risk transmission in the medical system at the beginning of the outbreak not only revealed the staff's failure to correctly diagnose the epidemics risk but also reflected a serious lack of availability of related medical protection materials. Therefore, after the outbreak of the COVID-19 epidemic, the Chinese government organized a large number of medical protection substance companies to expand production, and all walks of life in society actively organized the donation of related medical protection materials to meet the urgent needs for medical protection materials.

#### Social System Risk Transmission Path

The risk of sudden infectious epidemic outbreaks is not limited to the medical system. The risk of the COVID-19 epidemic spilled over from the medical system to the social system and has had a serious negative impact on the entire society. Studies have found that the COVID-19 epidemic in China will have a wide range of negative consequences for the economy (55). The virus's uncertainty has disrupted global trade and supply chains, depressed asset prices and forced multinationals to make difficult decisions with limited information. Many companies have evacuated their foreign employees from cities and temporarily stopped business activities. In terms of trade, strict travel restrictions imposed by different urban communities in Wuhan and Hubei are expected to have a chain effect across China and even around the world. Among industries that are negatively affected, retail, tourism, and hotel industries may be the most affected (56). The social system risk transmission of a sudden infectious epidemic is mainly manifested as an impact on social production and social life, and it is continuously transmitted upstream and downstream along the social production chain and life chain. On the one hand, as far as social production is concerned, the production activities of all walks of life in the entire society represent a highly relevant and dynamic, interlocking production chain and supply chain (57). Affected by the COVID-19 epidemic, many enterprises requiring staff, materials, and logistics are affected by the epidemic. This has led to, difficulty in orderly advancing the resumption of production. In addition, the outbreak of the epidemic will also affect social demand and will have a restraining effect (58). On the other hand, in terms of social life, in order to cope with the spread of the epidemic, the Chinese government has closed public transportation in many cities. The government issued recommendations to maintain social distance and recommended that people stay at home as much as possible, and schools have postponed their opening date. In addition, many migrant workers' return to work has also been greatly affected. From these, we find that the lives of people in the entire society have been greatly affected.

#### The Social Psychological Risk Transmission Path

The social psychological risk transmission path refers to the process by which the epidemic produces ripple effects on the psychological level of people and makes the public suffer from negative emotions such as fear, anxiety, tension, and distress. It is not limited to diagnosed patients and the general public, as it also negatively impacts healthcare workers (59). The outbreak of the COVID-19 epidemic in China was sudden, rapid, and extremely uncertain, and there is currently no cure or drug treatment. Moreover, we found that in society, there is a fear of Hubei people. The people's panicky emotions have also led to the behavior in which lifestyle supplies are snapped up. In addition, many medical staff are also under extremely strong psychological pressure and are experiencing negative emotions due to their high work pressure. These fears and nervous psychological emotions will often spread to other people around them. Consequently, the individual psychological negative emotions eventually develop into group psychological negative emotions.

## Conclusion

### Conclusions

From SARS and Zika virus to the outbreak of Ebola, human society has faced increasingly more sudden infectious epidemics. Once the sudden infectious epidemic breaks out, it not only causes casualties, but also have a great negative impact on social and economic activities. The breakout of COVID-19 epidemic in China in December 2019 has spread rapidly, and led to the increasingly diagnosed patients. This epidemic even now encompasses the globe, and the number of diagnoses and deaths is still increasing. Although there are many research achievements in the academic community on sudden infectious epidemic, some scholars also have paid attention to the risk early warning of sudden infectious epidemics and evaluated the social losses caused by the epidemic (60), but the existing studies rarely analyze the risk transmission law of the epidemic. This article takes the COVID-19 epidemic in China as an example, uses the Chinese media's report on the epidemic as the data sources, and uses grounded theory to develop a theoretical model of the risk transmission mechanism about the sudden infectious epidemic (see Figure 2). This study found that there are six basic elements: the risk source, the risk early warning, the risk transmission path, the risk transmission victims, the risk transmission inflection point, and the end of risk transmission. After a sudden infectious epidemic breaks out, there are three risk transmission paths, namely, medical system risk transmission path, social system risk transmission path and psychological risk transmission path, and these three paths present coupling structure. These research findings are the biggest theoretical contribution of this paper.

### Policy Implications

In the outbreak of the COVID-19 epidemic in China, this paper found that the spread of the risk is a process of step by step and dynamic evolution that continuously conduct its risks to the entire society through different paths. Based on the research findings, it is proposed that the emergency management of sudden infectious epidemics should be strengthened from three aspects: the control of the risk source, the establishment of an efficient and scientific risk early warning mechanism and the blocking of the risk transmission path. Firstly, scientific and efficient controlling the infectious epidemic sources have important roles in preventing the occurrences of accidents. The risk sources of sudden infectious epidemics can be divided into natural risk sources and man-made risk sources. For man-made risk sources, it is necessary to comprehensively strengthen the management of relevant biological laboratories to prevent the leakage of risk sources. For natural risk sources, it is necessary to strengthen ecological environmental protection and severely crack down on illegal wildlife trading. Secondly, establish a scientific event risk early warning system. The occurrences of sudden public health events often have corresponding risk signals at the beginning. However, due to multiple reasons such as insufficient understanding of the epidemics, the early warning emergency mechanism failed, then the risk transmission of the epidemics are not cut off in time at the beginning of the outbreak, resulting the risk epidemic spread. Due to the failure of the public health early warning mechanism in this COVID-19 epidemic, the conclusion of the outbreak was “preventable and controllable, and no obvious human-to-human transmission phenomenon” was reached at the beginning of the outbreak, which miss the best controlling time, leading the epidemic spread. Therefore, establishing a scientific emergency early warning mechanism for sudden public health events is the key to achieve rapid response, rapid decision-making, and rapid management of sudden public health events, and to control the epidemic risk in the initial state. Specifically, the most important thing is to increase the frequency of nucleic acid tests for employees in high-risk areas and high-risk industries, so that once someone is infected, they can be found in time. Temperature tests should also be conducted in densely populated areas, and once abnormal temperature is found, they can be handled immediately. In addition, it is necessary to strengthen the closed management of fever clinics in hospitals, and nucleic acid tests should be carried out on patients who enter hospitals for cold treatment. Finally, blocking the risk transmission paths of sudden infectious epidemics. The hazards of the sudden infectious epidemics continue to spread through its risk transmission paths. Therefore, blocking the risk transmission paths of the epidemics is conducive to reducing the social harm caused by the epidemics after the outbreak. The risk transmission paths of sudden infectious epidemics include medical system risk transmission path, social system risk transmission path and psychological risk transmission path. These three risk transmission paths are mutual influence. Therefore, comprehensive measures must be taken to block risk transmission n paths of the sudden infectious epidemics. Specifically, the most important thing is to speed up vaccine research and production so that more people can be vaccinated more quickly, which is the most important measure to stop the spread of the disease. In addition, it is also necessary to encourage people wear masks when going out and reduce the number of people gathering together. Psychological intervention should be strengthened for medical workers and patients confirmed to alleviate their panic through psychological counseling services.

### Research Limitations and Suggestions for Future Research

The number of confirmed cases of the COVID-19 epidemic has exceeded 5 million in the world. As an exploratory study, this paper takes 20 reports on COVID-19 from Chinese media as the original data, and use grounded theory to analyze these original data, so as to find the risk transmission law of the COVID-19. The limitations of this research lies in the fact that the original data of the epidemic report is only from the reports by media. The research found in this article requires scholars to use other research methods and data to further verify from other perspectives. In addition, it is necessary to specifically dig the Research Topics of the medical system risk transmission path, social system risk transmission path and psychological risk transmission path during the process of sudden infectious epidemics. For example, to analyze the negative impact of the sudden infectious epidemics on the international aviation industry and the tourism industry, the psychological changes and intervention measures of the governments in the epidemic, for these issues we need to conduct further detailed research in the future.

## Data Availability Statement

The original contributions presented in the study are included in the article/supplementary material, further inquiries can be directed to the corresponding author/s.

## Author Contributions

XD drafted the manuscript. ZZ conceptualized and designed the study. WZ contributed to materials and analysis. All authors contributed to the article and approved the submitted version.

## Funding

This research was supported by National Social Science Fund KeyProject of China (#18AZD004) and Graduate Education Innovation Grant Program in Central China Normal University (grant no. 2020CXZZ011).

## Conflict of Interest

The authors declare that the research was conducted in the absence of any commercial or financial relationships that could be construed as a potential conflict of interest.

## Publisher's Note

All claims expressed in this article are solely those of the authors and do not necessarily represent those of their affiliated organizations, or those of the publisher, the editors and the reviewers. Any product that may be evaluated in this article, or claim that may be made by its manufacturer, is not guaranteed or endorsed by the publisher.

## Appendix

**Appendix A1:** The description of 20 news reports.

**No**.	**Title**	**Source**	**Date**	**No**.	**Title**	**Source**	**Date**
1	Shanghai allocated epidemic support funds to cinemas in the first half of the year	*www.thepaper.cn*	26 February 2020	11	What can we do for the lack of medical supplies in Wuhan?	*Sanlian Lifeweek*	25 January 2020
2	Several zoos have been closed due to the COVID-19 outbreak	www.thepaper.cn	24 January 2020	12	Why did the COVID-19 in Wuhan not attract more attention until today?	*Sanlian Lifeweek*	27 January 2020
3	Public transport operations resumed in the country's second worst hit city	www.guancha.cn	21 March 2020	13	COVID-19 prevention and control has entered a new stage	Caixin.com	7 February 2020
4	The economy weathered the COVID-19 outbreak in January–February	www.guancha.cn	16 March 2020	14	The COVID-19 epidemic affects the housing market	Caixin.com	1 February 2020
5	The Ministry of Human Resources and Social Security responded to the impact of COVID-19 on employment	www.guancha.cn	24 February 2020	15	Critical moment of COVID-19 prevention and control: Wuhan medical staff and beds still need to be supplemented	Caixin.com	17 February 2020
6	She didn't have to speak. We all felt the same way	*Sourthernweekly*	2 January 2020	16	The economy has been hit by the epidemic, but it has withstood the test	*ChinaDaily*	17 March 2020
7	Full record of the COVID-19	*Sourthernweekly*	6 February 2020	17	More medical teams have been sent to Hubei province	*China Daily*	10 February 2020
8	Hubei people are not viruses and should not be shut out	*Sourthernweekly*	27 January 2020	18	A quick overview of the epidemic yesterday and this morning	*Xinhuanet*	2 February 2020
9	Covid-19 calls for psychological Intervention: Dealing with panic is also a major task	*Sourthernweekly*	27 January 2020	19	The National Banking Association is acting to rally financial forces to fight the epidemic.	*Xinhuanet*	19 February 2020
10	Catering industry under COVID-19	*Sanlian Lifeweek*	10 February 2020	20	Community workers are under great pressure to fight the epidemic and urgently need help	*Xinhuanet*	12 February 2020
